# Hepatic dysfunction in patients who received acute DeBakey type I aortic dissection repair surgery: incidence, risk factors, and long-term outcomes

**DOI:** 10.1186/s13019-021-01676-8

**Published:** 2021-10-10

**Authors:** Zhigang Wang, Min Ge, Cheng Chen, Lichong Lu, Lifang Zhang, Dongjin Wang

**Affiliations:** 1 Department of Cardio-Thoracic Surgery, Affiliated Drum Tower Hospital, Medical School of Nanjing University, Zhongshan Road 321, Nanjing, 210008 China; 2 Department of Psychiatry, The First Affiliated Hospital, Zhengzhou University, Zhengzhou, China

**Keywords:** Hepatic dysfunction, DeBakey type I aortic dissection, Model for end-stage liver disease score, Multivariate analysis

## Abstract

**Background:**

Hepatic dysfunction (HD) increases the morbidity and mortality rates after cardiac surgery. However, few studies have investigated the association between HD and acute DeBakey type I aortic dissection (ADIAD) surgery. This retrospective study aimed to identify risk factors for developing HD in patients who received acute type I aortic dissection repair and its consequences.

**Methods:**

A total of 830 consecutive patients who received ADIAD surgery from January 2014 to December 2019 at our center were screened for this study. The End-Stage Liver Disease (MELD) score more than 14 was applied to identify postoperative HD. Logistic regression model was applied to identify risk factors for postoperative HD, Kaplan–Meier survival analysis and Cox proportional hazards regression assay were conducted to analyze the association between HD and postoperative long-term survival.

**Results:**

Among 634 patients who eventually enrolled in this study, 401 (63.2%) experienced postoperative HD with a 30-Day mortality of 15.5%. Preoperative plasma fibrinogen level (PFL) [odds ratio (OR): 0.581, 95% confidence interval (CI): 0.362–0.933, *P* = 0.025], serum creatinine (sCr) on admission (OR: 1.050, 95% CI 1.022–1.079, *P* < 0.001), cardiopulmonary bypass (CPB) time (OR: 1.017, 95% CI 1.010–1.033, *P* = 0.039), and postoperative mechanical ventilation (MV) duration (OR: 1.019, 95% CI 1.003–1.035, *P* = 0.020) were identified as independent risk factors for developing postoperative HD by multivariate analyses. In addition, the Kaplan–Meier analysis indicated that the long-term survival rate was significantly different between patients with or without postoperative HD. However, the hazard ratios of long-term survival for these two groups were not significantly different.

**Conclusions:**

HD was a common complication after ADIAD surgery and associated with an increasing 30-Day mortality rate. Decreased PFL, elevated sCr, prolonged CPB duration, and longer postoperative MV time were independent risk factors for postoperative HD.

## Introduction

Acute type A aortic dissection (ATAAD) is a life-threatening condition requires immediate surgical repair. The hourly mortality rate of ATAAD can be as high as 1% to 2% after symptoms onset before surgical intervention [1]. Even though with the advancing techniques, the successful rate of ATAAD surgery has been increasing, the 30-Day mortality is still relatively high with approximately 10% to 20% according to recent comprehensive studies [2, 3]. Acute DeBakey type I aortic dissection (ADIAD) is a severe subset of ATAAD and demands a more challenging surgical procedure [4]. Most ADIAD operation are performed in emergency, and prolonged cardiopulmonary bypass (CPB) time and multiple transfusions are often occurred. What’s more, the short-term malperfusion complications and long-term adverse aortic events arising after surgical treatment of ADIAD are considered to be high because of the wide presence of a false lumen [5, 6].

Hepatic dysfunction (HD) is a common complication after cardiothoracic surgery which often result in increasing mortality. It has been estimated that the incidence of postoperative HD after cardiovascular surgeries ranges from 21.9 to 60.9% [7–9]. The most important criteria of HD had been the Child-Turcotte-Pugh (CTP) classification for the past 30 years which has been challenged recently by the model of End-Stage Liver Disease (MELD) score. Both of these two criteria have been applied to examine the association between HD and ADIAD surgery yet limited by the small cohorts of fewer than 100 patients with a definite diagnoses of liver cirrhosis [10–12]. Recently, several reports proved that MELD is a better assessment tool to predict postoperative mortality than CTP in patients received cardiothoracic surgeries, as it does not include subjective variables [8, 13].

Even though multiple studies have been conducted to examine the association between HD and general cardiothoracic surgeries, the association between HD and ADIAD has not been specifically studied. In this retrospective analysis, we applied the MELD criteria to identify the risk factors for postoperative HD and examine its influence on long-term prognosis in a relatively large cohort of ADIAD patients who received aortic dissection surgery.

## Methods and materials

### Study population

This study was approved by the Ethics Committee of the Nanjing Drum Tower Hospital. The individual patient consent was waived considering the nature of this study. A total of 830 medical records of patients who received ADIAD surgery within 14 days of symptom onset at our center between January 2014 and December 2019 were retrospectively screened. Patients who were on renal replacement therapy before surgery (n = 26) were excluded because its influence on serum creatinine (sCr). In addition, patients who died during or within 24 h after surgery (n = 18) were excluded. Patients accompanied with severe alcoholic or viral liver disease (n = 18) were also excluded. Considering that the MELD score includes international normalized ratio (INR) in the formula, patients who received warfarin before or after surgery (n = 129) were excluded from this study. 5 patients with incomplete data were excluded from the study as well. Patients’ general health status was followed up once every year by telephone since 2014. The date and cause of deaths were collected during the follow up if occurred. AKI was defined by the Kidney Disease Improving Global Outcome (KDIGO) classification by the changes in sCr.

### Diagnostic criteria for postoperative HD

The MELD score was calculated using the standard formula published by Mayo Clinic College of Medicine: MELD = 11.2 * In (INR) + 3.78* In (total bilirubin [mg/dL]) + 9.57* In (creatinine [mg/dL]) + 6.43* (*cause*). Where *cause* equaled 0 for bilious or alcoholic liver disease and 1 for otherwise [14]. Any variable with a value less than 1 was assigned a value of 1 to avoid negative scores. The MELD score was recorded daily for a week after the surgery during which the postoperative HD was determined based on the highest MELD score. Previous studies set the cutoff point as 12 or 13 for HD diagnosis after different surgical procedures [8, 10, 11]. Considering ADIAD surgery is more complicated than other cardiac surgeries, the cutoff value of 14 was selected to define postoperative HD in this study, which was consistent with a previous study [9]. Patients were then classified into HD group (≥ 14) and non-HD group (< 14).

### Statistical analysis

Categorical variables were presented as frequencies with percentages. Continuous variables were presented as means ± standard deviation or median with interquartile range (IQR). The χ^2^ tests or Fisher exact test was applied for categorical variables and the *t*-test was applied for normally distributed continuous variables whereas the Mann–Whitney *U*-test was used for non-normally distributed variables. Multivariate logistic regression analysis was conducted to identify independent risk factors for postoperative HD. For regression analysis, all variables with a *p*-value less than 0.50 in univariate analysis were included in the model. A stepwise enter method was used to introduce variables to the final models. A receiver-operating characteristic (ROC) curve analysis was performed to detect the best cut-off value of the predictors for postoperative HD. Crude survival was estimated with the Kaplan–Meier method. Differences in survival between groups were further analyzed with log-rank tests. Cox proportional hazard analysis was applied to identify independent factors that affect long-term survival. For Cox regression analysis, all variables with a *p*-value less than 0.20 identified in univariate analysis were included in the model. A stepwise enter procedure was used to introduce variables to the final models. A *p *value less than 0.05 was considered statistically significant. All analyses were conducted with SPSS version 25.0 (IBM Corp, Armonk, NY, USA).

## Results

A total of 634 patients were eventually included in the study with a median age of 52.5 years old (range 24–85 years), and 480 (75.7%) of these patients were men. Our data suggested that a total of 401 patients (63.2%) developed postoperative HD. Patient clinical characteristics and preoperative variables associated with MELD score were presented in Table 1. The time from symptom onset to surgery and liver blood supply condition were similar between HD and non-HD groups. For serum laboratory test results, there were significant differences between patients with or without HD in terms of white blood cell, platelet, fibrinogen, troponin T, D-dimer, albumin, creatinine, blood urine nitrogen, INR, total bilirubin, alanine transaminase, and aspartate transaminase upon admission. For the operative parameters, CPB duration and aortic cross-clamp time were significantly different between the two groups. Furthermore, we discovered that patients with postoperative HD often resulted in unfavorable outcomes, including more frequent dialysis requirement, postoperative acute kidney injury, drainage volume 24 h after surgery, tracheostomy rate, longer intensive care unit stay time, and worse 30-Day mortality (Table 2).

**Table 1:** Comparison of preoperative variables

Variables	Total (n = 634)	MELD < 14 (n = 233)	MELD ≥ 14 (n = 401)	*P* Value^a^
Demographic data				
Age (year)	53.0 ± 12.4	53.4 ± 12.1	52.8 ± 12.6	0.348
Male (%)	480 (75.7)	161 (69.1)	319 (79.6)	**0.003**
BMI (kg/m^2^)	26.4 ± 4.7	26.1 ± 4.3	26.5 ± 4.9	0.243
Medical history				
Hypertension (%)	492 (77.6)	178 (76.4)	314 (78.3)	0.578
Diabetes mellitus (%)	15 (2.4)	7 (3.0)	8 (2.0)	0.420
Previous cardiac surgery (%)	30 (4.7)	11 (4.7)	19 (4.7)	0.992
Previous cardiovascular disease (%)	20 (3.2)	6 (2.6)	14 (3.5)	0.525
Cerebrovascular disease (%)	24 (3.8)	10 (4.7)	14 (3.2)	0.347
MELD score	16.8 (11.9, 22.4)	11.2 (9.4, 12.5)	20.6 (17.1, 26.4)	**< 0.001**
Time from onset to surgery (h)	16.8 ± 5.2	17.4 ± 7.2	16.3 ± 6.4	0.644
Pericardial tamponade (%)	110 (17.4)	37 (15.9)	73 (18.2)	0.456
Involving the celiac trunk (%)	134 (21.1)	47 (20.2)	87 (21.7)	0.676
Involving the superior mesenteric artery (%)	120 (18.9)	43 (18.5)	78 (19.2)	0.703
Preoperative laboratory data				
WBC (10^9^/L)	11.5 (8.8, 14.3)	11.0 (8.7, 13.4)	11.7 (8.8, 14.9)	**0.038**
Hemoglobin (g/L)	124.2 ± 24.6	127.2 ± 20.7	122.4 ± 26.5	0.102
PLT (10^9^/L)	153.2 ± 94.5	166.9 ± 126.5	145.4 ± 68.7	**< 0.001**
Fibrinogen (g/L)	2.2 (1.6, 3.0)	2.5 (1.8, 3.5)	2.0 (1.5, 3.0)	**< 0.001**
Triglyceride (mmol/L)	1.1 (0.7, 1.5)	1.1 (0.7, 1.5)	1.1 (0.8, 1.5)	0.841
CRP (mg/dl)	23.7 (4.9, 84.4)	22.0 (4.4, 80.0)	30.3 (5.8, 88.4)	0.148
TNT (ng/ml)	0.02 (0.01, 0.11)	0.01 (0.01, 0.03)	0.03 (0.01, 0.16)	**< 0.001**
D-dimer (ng/mL)	4.9 (2.7, 9.9)	4.2 (2.1, 7.5)	5.1 (3.0, 11.7)	**0.005**
Albumin (g/L)	36.8 ± 5.0	37.9 ± 4.1	36.2 ± 5.3	**0.001**
ALT (U/L)	25.1 (16.2, 44.5)	20.4 (15.1, 35.7)	29.0 (17.2, 52.3)	**< 0.001**
AST (U/L)	31.2 (21.0, 53.6)	26.0 (19.0, 41.3)	33.3 (22.0, 72.0)	**< 0.001**
Cr (μmol/L)	100.1 ± 65.7	68.2 ± 19.8	118.6 ± 75.2	**< 0.001**
Bun (mmol/L)	8.1 ± 3.6	6.7 ± 2.4	9.0 ± 3.9	**< 0.001**
Total bilirubin (μmol/L)	15.3 (11.2, 22.8)	13.7 (10.5, 20.0)	16.6 (11.8, 23.9)	**0.001**
INR	1.08 (1.00, 1.20)	1.05 (0.97, 1.12)	1.13 (1.03, 1.25)	**< 0.001**

Bold values indicate significance at *p* < 0.05

Data presented as n (%), median (IQR), or means ± standard deviation

*BMI* body mass index, *WBC* white blood cell, *PLT* platelet, *CRP* c-reactive protein, *TNT* troponin T, *ALT* alanine transaminase, *AST* aspartate transaminase, *Cr* creatinine, *Bun* blood urea nitrogen, *INR* international normalized ratio

^a^*P* values indicate differences between HD patients and non-HD patients. *P* < 0.05 was considered statistically significant

**Table 2:** Comparison of operative and postoperative variables

Variables	Total (n = 634)	MELD < 14 (n = 233)	MELD ≥ 14 (n = 401)	*P* Value^a^
TAR (%)	359 (56.6)	129 (55.4)	230 (57.4)	0.626
CABG (%)	28 (4.4)	5 (2.1)	23 (5.7)	**0.034**
CPB time (min)	225.9 ± 62.8	213.0 ± 52.4	233.5 ± 67.0	**< 0.001**
Aortic cross-clamp time (min)	155.6 ± 51.9	147.4 ± 46.2	160.4 ± 54.5	**0.004**
DHCA time (min)	30.7 ± 12.4	30.3 ± 11.2	30.9 ± 13.0	0.922
Drainage volume 24 h after surgery (ml)	545.0 (320.0, 892.5)	450.0 (287.5, 670.0)	600.0 (350.0, 947.5)	**< 0.001**
Re-exploration for bleeding (%)	23 (3.6)	10 (4.3)	13 (3.2)	0.495
Dialysis (%)	95 (15.0)	0 (0)	95 (23.7)	**< 0.001**
AKI (%)	366 (57.7)	79 (33.9)	287 (71.6)	**< 0.001**
Mechanical ventilation time (hour)	17.9 (11.4, 44.3)	14.3 (9.6, 23.0)	23.8 (13.6, 62.0)	**< 0.001**
Stroke (%)	59 (9.3)	20 (8.6)	39 (9.7)	0.633
Paraplegia (%)	22 (3.5)	6 (2.6)	16 (4.0)	0.348
Tracheostomy (%)	28 (4.4)	4 (1.7)	24 (6.0)	**0.012**
Deep sternal wound infection (%)	11 (1.7)	1 (0.4)	10 (2.5)	0.063
ICU stay time (day)	5.0 (3.0, 8.0)	4.0 (3.0, 5.0)	6.0 (4.0, 10.0)	**< 0.001**
Hospital stay time (day)	21.6 ± 12.0	19.0 ± 7.6	23.1 ± 13.7	**< 0.001**
30-Day mortality (%)	69 (10.9%)	7 (3.0)	62 (15.5)	**< 0.001**

Bold values indicate significance at *p* < 0.05

Data presented as n (%), median (IQR), or means ± standard deviation

*TAR* total arch replacement, *CABG* coronary artery bypass graft, *CPB* cardiopulmonary bypass, *DHCA* deep hypothermic circulatory arrest, *AKI* acute kidney injury, *ICU* intensive care unit, *HD* hepatic dysfunction

^a^*P* values indicate differences between HD patients and Non-HD patients. *P* < 0.05 was considered statistically significant

As suggested in Table 3, the multivariate analysis indicated that preoperative plasma fibrinogen level (PFL) [odds ratio (OR): 0.581, 95% confidence interval (CI): 0.362–0.933, *P* = 0.025], preoperative sCr (OR: 1.050, 95% CI 1.022–1.079, *P* < 0.001), CPB time (OR: 1.017, 95% CI 1.010–1.033, *P* = 0.039), and postoperative mechanical ventilation (MV) duration (OR: 1.019, 95% CI 1.003–1.035, *P* = 0.020) were independent risk factors for developing postoperative HD.

**Table 3:** Multivariate analysis of risk factors for postoperative HD

Variables	OR	95% CI	*P* value
Age	1.001	0.962–1.043	0.945
Male	0.712	0.189–2.684	0.616
BMI	1.016	0.913–1.131	0.769
Diabetes mellitus	0.486	0.027–8.810	0.626
Cerebrovascular disease	1.346	0.078–23.186	0.838
Pericardial tamponade	1.698	0.294–9.802	0.554
WBC	0.957	0.882–1.039	0.298
Hemoglobin	1.001	0.975–1.029	0.915
PLT	1.009	1.000–1.019	0.058
Fibrinogen	0.581	0.362–0.933	**0.025**
CRP	0.997	0.984–1.009	0.590
TNT	1.065	0.219–5.177	0.938
D-dimer	1.036	0.986–1.087	0.160
Albumin	0.956	0.851–1.074	0.446
ALT	0.995	0.978–1.013	0.577
AST	1.015	0.991–1.040	0.220
Cr	1.050	1.022–1.079	** < 0.001**
Bun	1.137	0.899–1.439	0.284
Total bilirubin	1.042	0.996–1.091	0.076
INR	1.944	0.774–4.880	0.157
CABG	0.123	0.003–4.385	0.250
CPB time	1.017	1.010–1.033	**0.039**
Aortic cross-clamp time	0.994	0.977–1.011	0.480
Drainage volume 24 h after surgery	1.001	1.000–1.002	0.069
Mechanical ventilation time	1.019	1.003–1.035	**0.020**

Bold values indicate significance at *p* < 0.05

*HD* hepatic dysfunction, *BMI* body mass index, *WBC* white blood cell, *PLT* platelet, *CRP* c-reactive protein, *TNT* troponin T, *ALT* alanine transaminase, *AST* aspartate transaminase, *Cr* creatinine, *Bun* blood urea nitrogen, *INR* international normalized ratio, *CABG* coronary artery bypass graft; CPB, cardiopulmonary bypass

*P* < 0.05 was considered statistically significant

**Fig. 1:**
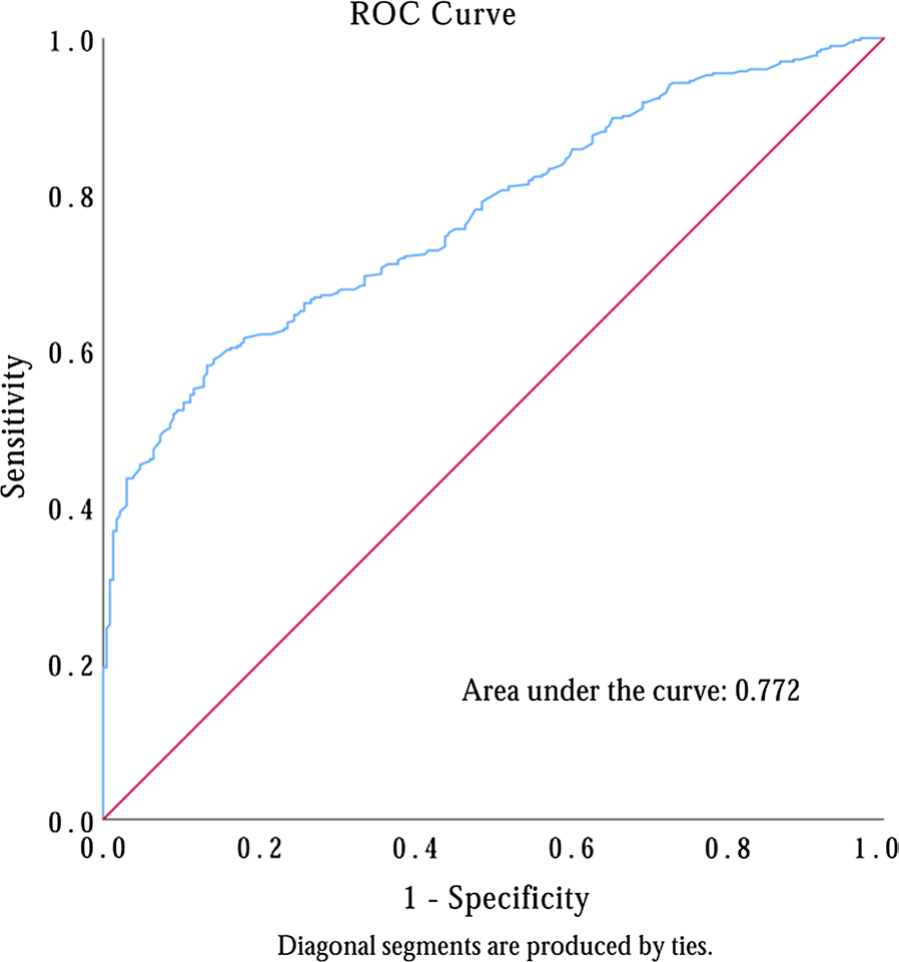
Receiver operating characteristics curve for determination of the cut-off for prognostic serum creatinine value upon admission in predicting postoperative hepatic dysfunction in DeBakey type I aortic dissection

In addition, to better determine the predictive value of PFL and sCr. The ROC curves were plotted and the cutoff values of PFL and sCr levels upon admission for preoperative HD were 2.25 g/L and 88.85 μmol/L, respectively. These values were associated with a sensitivity of 57.7% and a specificity of 60.9% for PFL and of 58.1% and 86.7% for sCr, respectively. In addition, the AUC for PFL was 0.606 (95% CI 0.561–0.651, *P* < 0.001) and 0.772 (95% CI 0.737–0.807, *P* < 0.001) for sCr, respectively (Fig. 1).

**Fig. 2:**
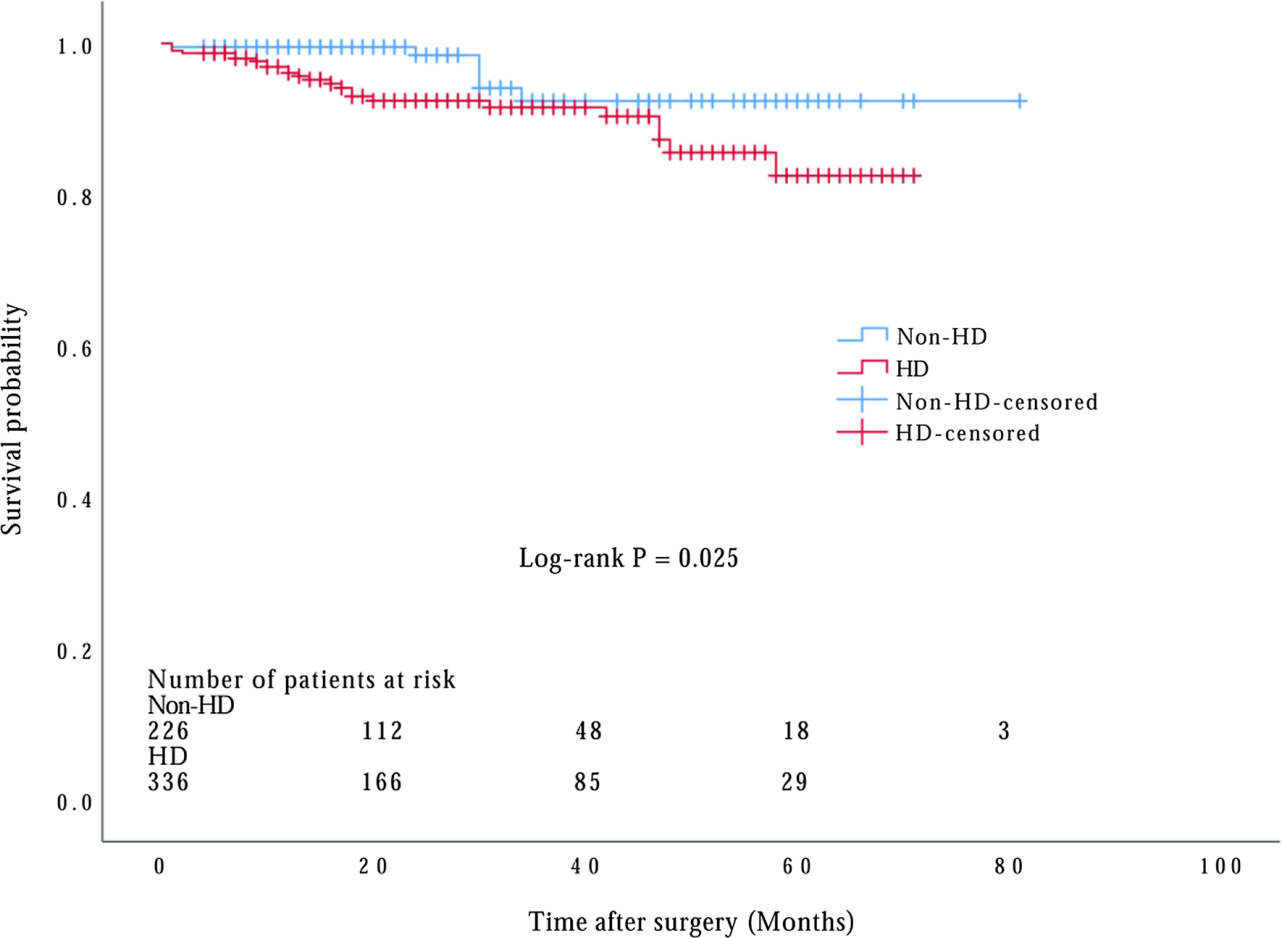
Kaplan–Meier survival curve of postoperative hepatic dysfunction in all patients with acute DeBakey type I aortic dissection treated with surgical repair

65 patients (16.2%) in the HD group and 7 patients (3.0%) in the non-HD group died during the hospitalization period. By December 2020, 562 patients had been followed up with a median of 20 months. 38 of 562 (6.8%) patients were lost from the cohort and excluded from the survival analysis. A total of 6 patients dead in the non-HD group and 24 in the HD group during follow-up period. A significant overall difference was indicated between two groups by the Kaplan–Meier survival curves (Fig. 2; *P* = 0.025 by log-rank test), whereas after adjusting for confounders, the hazard ratios for postoperative HD (1.150, 95% CI 0.295 to 4.481, *P* = 0.841) was not significantly different compared to non-HD patients (Table 4).

**Table 4:** Hazard ratios for long-term survival

Variables	Hazard ratio	95% CI	*P* value
Age (≥ 65 year)	3.026	0.956–9.579	0.060
Pericardial tamponade	2.689	0.715–10.120	0.143
Postoperative AKI	9.502	1.143–79.022	**0.037**
Postoperative HD	1.150	0.295–4.481	0.841
Prolonged MV time (≥ 48 h)	7.039	2.413–20.530	**< 0.001**

Bold values indicate significance at *p* < 0.05

*AKI* acute kidney injury, *HD* hepatic dysfunction, *MV* mechanical ventilation

*P* < 0.05 was considered statistically significant

## Discussion

In this study, we examined the incidence and risk factors of developing postoperative HD in patients who received ADIAD surgery. Our data suggested that among all enrolled 634 patients, 401 patients (63.2%) developed postoperative HD. Multivariate logistic regression analysis revealed that decreased PFL and elevated sCr on admission, longer CPB duration, and longer MV time were independent risk factors for postoperative HD. The present study also indicated a clear trend that patients with postoperative HD had a lower survival rate, although the hazard ratios was not significantly different compared to patients without HD. The strength of the present study is that this was the first study aimed to identify the risk factors for and long-term outcomes of postoperative HD in a relatively large cohort which also excluded the influence of warfarin treatment.

The incidence of postoperative HD in our study was similar to that found in a previous study [9] which showed a incidence of HD of 60.9% among 215 patients who received ATAAD surgery with deep hypothermic circulatory arrest. Liu and colleagues [15] reported that early postoperative HD incidence was 8.7% after ATAAD surgery, while another study showed an HD incidence of 1.5% postoperatively among patients with descending thoracic and thoracoabdominal aortic dissection [16]. However, these studies relied on biochemical markers to evaluate liver function rather than a comprehensive evaluation.

Our data suggested that the 30-Day mortality in patients with HD was 15.5% (62 of 401 patients), which was comparable to a previous study focused on thoracic aortic surgery (16.8%) [9]. It has been known that postoperative HD is associated with worse disease outcomes such as 30-Day mortality, morbidity, and increasing cost [7, 8]. However, these studies did not examine the association between postoperative HD and long-term survival. The present study showed that patients with postoperative HD have a significantly lower long-term survival rate compared to patients without HD. However, after adjusting for confounders, the hazard ratios for postoperative HD was not significantly higher compared to patients without HD. It might be that the HD occurred after surgery was temporary and did not result in chronic liver disease. Therefore, identifying risk factors and preventing postoperative HD is critical in improving short-term prognosis. For long-term survival, more studies and longer follow-up are needed to confirm the reproducibility of these results.

The results of this study demonstrated that preoperative sCr was a risk factor for postoperative HD, which implied that presence of impaired renal function contributed to developing deranged liver function tests. As the OR for preoperative sCr to predict postoperative HD is only 1.050, which imply that preoperative renal function is not a strong risk factor that affect postoperative HD. A previous study showed that preoperative sCr > 133 μmol/L was a risk factor for postoperative HD in patients undergoing ATAAD operations [15], which was consistent with our results. Acute liver dysfunction is associated with renal insufficiency, whose mechanism may be related to activation of the hepatorenal reflex [17, 18].

The logistic regression model identified lower PFL as an independent risk factor for developing HD. Decreased fibrinogen level upon hospital admission within 24 h after symptom onset has been demonstrated as a powerful predictor of mortality in patients with ATAAD [19, 20], as fibrinogen is a very common substance in the coagulation system and could lead to activation of the coagulation cascade [21]. It has been proved that low PFL is a risk factor for postoperative blood loss [22, 23]. Several possible mechanisms might explain the association between low fibrinogen level and postoperative HD. Fibrinogen is synthesized and secreted by the liver and participates in the thrombosis. Previous studies indicated that the level of coagulation and fibrinolytic activation was proportionally related to the anatomic extent of the dissection [24]. Intimal tearing in aortic dissection induces subendothelial element exposure and tissue factor release which further activates the coagulation cascade. This process consumes large amounts of fibrinogen and results in a decreased serum level. It has been hypothesized that lower PFL indicates greater extent injury of the aorta and more severely impaired liver function. On the other hand, it has been proposed that PFL as a surrogate marker for consumption coagulopathy in patients with dissecting aorta which is associated with increasing risk of transfusion, complications, and fatality [25]. Large prospective cohort studies should be performed to further explore the role of fibrinogen in TAAD treatment.

In addition to fibrinogen level, longer CPB duration was identified as another risk factor for HD, as has been demonstrated previously [15]. It has been reported that the blood volume in liver arteries decreases by 20–25% during CPB [26]. The decreased liver perfusion often results in liver damage. Furthermore, surgical procedure and CPB could induce the release of inflammatory cytokines and endotoxins, which influences the hemodynamics, coagulation system, immune system, vascular resistance and permeability as well as platelet concentration and function [27]. Furthermore, hypothermia and nonpulsatile circulation during CPB are thought to contribute to hemodynamic deterioration that often lead to overwhelming protective mechanisms and synergistically exacerbate hypoxic liver damage [28]. However, another study reported that the aortic cross-clamp time, rather than CPB time, was associated with the developing of HD [9]. These controversies might be attributed to confounding factors in heterogeneous patient cohorts.

The postoperative MV time was identified as another risk factor for postoperative HD. Prolonged MV duration indicated an extended postoperative respiratory dysfunction. Previous study showed that the incidence of prolonged MV (≥ 48 h) was up to 44.5% after ADIAD surgery [29]. Previous studies identified that prolonged extubation substantially increased the risk of developing postoperative acute kidney injury in cardiac surgical patients [6, 30, 31]. Our findings suggested that the duration of postoperative positive pressure ventilation was an important and previously under estimated risk factor for developing HD after ADIAD surgery that was consistent with a previous study which identified postoperative respiratory dysfunction as a risk factor for HD in patients who received ATAAD surgery [9]. MV can reduce cardiac output, blood pressure, effective circulating blood volume, and ultimately liver perfusion. It is believed that prolonged MV time not only induces hemodynamic disturbance but also aggravates systemic inflammatory reaction through mechanical damage to the lung [32]. It has been well known that MV is an important risk factor of causing and aggravating hepatic function damage in critical ill patients even after successful resuscitation [33].

## Limitations

This study had some limitations. Firstly, although the number of patients was relatively large compared with other studies, this study was conducted in a single center and might not be representable for the general population. Secondly, because of the emergent nature of the disease, complete demographic data were absent for some patients. Finally, our surgical technique has been advanced over the study period, and the results in this study might be influenced by the involvement of different techniques and surgeons. Therefore, additional prospective multicenter studies are warranted to assess the prognostic significance of HD and establish the most effective strategies to prevent and treat postoperative HD in patients who received ADIAD surgery.

## Conclusions

In conclusion, our data confirmed that the HD was a common complication among patients who received ADIAD surgery. The decreased PFL, elevated sCr, increased CPB duration, and prolonged MV time were identified as independent risk factors for postoperative HD.

## Data Availability

Data sharing in the current study are available from the corresponding author on reasonable request.

## Abbreviations

HD: Hepatic dysfunction
ADIAD: Acute DeBakey type I aortic dissection
MELD: Model of end-stage liver disease
PFL: Preoperative fibrinogen level
CPB: Cardiopulmonary bypass
MV: Mechanical ventilation
OR: Odds ratio
CI: Confidence interval
